# 

**Published:** 1889-06

**Authors:** 


### GENERAL INDEX—VOL. IV.

Anatomical Changes in Spinal Cord .................... 12
Austin District Medical Society....20, 127, 165, 301, 337, 373, 424
Andidote to Serpent Venom............................ 34
A New Instrument of Texas Invention............... ... 36
Another new Journal.......... ........................ 36
American International Congress of Medical Jurisprudence 43
A new Source of Revenue to the Medical Publisher...... 81
An Unfortunate Criticism and a threatened Boycott...... 84
American Association of Obstetricians.................. 166
After the Quacks....................................... 166
A Sensation in Medical Circles......................... 179
A Suggestion for the Good of the Cause................ 217
Appearance and Behavior of the Pupil in Health and in Disease ................................ ........ 237
Apoplexy ................................................ 241
A Question of Ethics: Answer wanted.................. 266
An Important Decision affecting Texas Doctors.......... 314
Acrostic on (Death of) Dr. Gregg...................... 315
Address of President Paine to the Members T. S. M. A...334, 341
American Educational Journalism ....................... 374
A State Board of Health for Texas...................... 379
A Good Investment of Money.... ...................... 380
An Insult to the Profession ........................... 389
Arbor Day in Texas .................................... 390
Absence of the Uterus and Malformation of Vagina and Uterus ................................ ............ 402
A Public Trial of Surgical Skill........................ 419
American Medical Association.......................... 492
Book Notices and Reviews—
Applied Anatomy of the Nervous System............ 231
Atlas of Venereal and Skin Diseases ..................39, 352
Annual of Universal Medical Science................230. 351
Case of Frederick III, The........................ 429
Diseases of Women, Hewitt........................ 229
Hand Book, Materia Medica, Bowen................ 392
Hand Book of Phthisiology ........................ 430
Hygiene of the Skin............................... 39
Medical Diagnosis................................. 429
Medical and Surgical Monographs.................. 430
Nervous Exhaustion, a Treatise on................. 429
Pathology and Treatment, Displacement of the Uterus 391

Pocket Medical Formulary .......................... 350
Proceedings Arkansas Medical Association .......... 231
Proceedings Florida Medical Association............ 231
Physicians' Pocket Reference....................... 350
Physicians' Pocket Day Book........................ 230
Physicians' Leisure Library........................ 392
Reference Hand Book of the Medical Sciences...... 40, 391
Treatise on Malarial Fevers........................ 351
Treatise on Diseases of Women...................... 351
Text Book Human Physiology......................... 229
Transactions Med. and Chirurgical Faculty Maryland. 352
Transactions Michigan State Medical Society........ 352
Transactions Missouri State Medical Society ........ 363
Transactions Pennsylvania Medical Society.......... 353
Transactions Texas State Med. Assoc'n....30, 32, 86, 134, 231
Transactions Several other State Associations, List... 353
Biography Contemporary Physicians of Texas...........421, 489
Biography of Dr. R. M. Swearingen, President T. S. M. A. 489
Barber's Itch, ℞ for................................ 426
Bichloride of Mercury in Diphtheria.................. 72
"Baffled Science"..................................... 34
Correspondence.....................37, 331, 260, 293, 119, 411
Complete Laceration of the Arm...................... 501
Convulsion from Injection of Cocaine ................ 37
Chronic Chills ...................................... 424
Central Texas Medical Society..................302, 389, 440
Cherokee County Medical Society.................128, 208, 371
Cullings from Contemporaries..............213, 72, 302, 374, 167
Circular Letter from President State Medical Association.. 19
Care and Management of Insane in Texas .............. 47
Correction in Dr. Smith's Paper...................... 125
Clinical Notes on Galvanism ......................... 201
Case of Septic Poisoning from Bed Sores.............. 205
Continued Fevers in Texas—Dr. West's Paper........... 514
Death of J. W. Lambert............................... 312
" Dr. A. C. White.................................... 278
" " W. R. Spalding .................................. 278
" " Bedell........................................... 278
" " Z. W. Baker...................................... 270
" " C. K. Gregg...................................... 178
" " Early Walton .................................... 177
" " J. T. Y. Jameson................................. 177
" " J. T. Howze...................................... 134
" " F. A. Tompkins ............................... 32, 87
" Mrs. T. C. Osborn .................................. 86
" Dr. J. M. Ross ..................................... 350
" " McKenzie ......................................... 344

" " H. W. Purnell .......... 33
" Mrs. Barton .......... 34
" Dr. Y. D. Harrington .......... 38
" " L. B. Johnson .......... 416
" Mrs. Lambert .......... 416
Diphtheria, Report of Five Cases .......... 366
Diphtheria .......... 433
Dr. Swearingen's Paper .......... 222
Dermoid Cyst, A .......... 60
Dermoid Cyst of the Coccygeal Region .......... 254
Double Ovariotomy at the Age of 69 .......... 68
Dr. Mayfield's Article .......... 75
Dr. Cunningham's Defense of the Hog .......... 276
Editorial .......39, 76, 129, 169, 217, 270, 305, 341, 377, 415, 488
Early Operation for Ptyregium .......... 161
East Line Medical Society .......... 26, 166
Equipment Texas Medical College .......... 86, 123
Extinction of Yellow Fever .......... 343
Exostosis Eburnea (Peeples) .......... 408
Flowers .......... 348
Fracture of Patella .......... 100
Foreign Correspondence .......... 119
Good Investment, A .......... 380
Galveston's Semi-Centennial .......... 493
Good Opening for a Physician .......... 87
Glycerole Compound in Internal Hemorrhoids .......... 62
Gadberry's Spleen Mixture .......... 424
Hysterectomy for Malignant Diseases of the Cervix .......... 196
Hemorrhoids, Fissure and Fistula in Ano .......... 109
How to treat Cramps in the legs .......... 74
How can Medical Societies be made more Profitable .......... 339
Incontinence in Males .......... 34
Ingrowing Nails .......... 425
Insanity, Nature and Causes of .......... 355
Influence of Malaria on Abdominal Viscera .......... 64
Insane in Texas, Care and Management of .......... 47
Ipecac in the Treatment of Disease .......... 74
Incompatibility of Antipyrin and Spirits of Nitre .......... 79
It Smells to Heaven .......... 169
In Memoriam, Dr. E. Mellou .......... 266
Is Pneumonia an Essential Fever? .......... 317
Important if True .......... 313
Important Decision affecting Texas Doctors .......... 413
Kettle and Pot .......... 29
Kansas Medical Society .......... 480
Limestone Co. Med. Society .......... 127
Lime in Urine .......... 439

Letter from Dr. R. P. Talley.......................... 331
" " Dr. Daniel Parker ........................ 293
" " Dr. Stovall ............................... 262
" " Dr. H. A. West ......................... 123
" " Scotland ................................ 119
" " Dr. T. J. Tyner.......................... 109
" " Dr. H. S. Robertson,...................... 411
" " Dr. R. H. Day............................ 412
" " Dr. Dock................................ 505
" " Dr. Eastland............................. 507
Mastoid Diseases .................................... 497
Medical News and Miscellany....33, 86, 133, 175, 225, 277, 311, 343, 420, 490, 529.
Married, Hill-Taylor ................................ 421
" Duprey-Lipscomb............................ 345
" Montgomery-Kelley ......................... 133
" Maxwell-King............................... 277
" Olive-Campbell ............................. 277
" Rucker-Bailey .............................. 491
" Tyner-Denison............................... 30
Medical Youth, Manhood, Age......................... 395
McKenzie and the German Doctors..................... 223
Medical Legislation ................................. 222
Miss. Valley Med. Association ...................... 72, 346
Membranous Croup in a Child 13 months old............ 31
Modern Treatment of Neuraligia....................... 29
Mistaken Notions of Hospitality...................... 131
Medical Department University Texas.................. 135
Management of the Cord............................... 290
Memphis Journal of the Medical Sciences.............. 338
New Orleans Med. and Surgical Journal................ 309
North Texas Med. Society.............................224, 493
Necrological......................................... 38
Original Communications....1, 47, 92, 139 186, 237, 281, 317, 355, 395, 433, 497.
Old Oaken Bucket, the ............................... 427
Ozone Water in Cancer................................ 345
Our Advertisers ..................................... 31
Obstruction of the Bowels............................ 117
Publishers' Notes.41, 89, 136, 182, 232, 280, 354, 393, 431, 494, 526
Proceedings Terrell Med. Society....................23, 297, 509
" Travis Co. Med. Society..................... 299
" Austin Distriet Med. Society ............... 20, 301
" Central Tex. Med. Society .................. 302
" Florida Med. Society ....................... 231
" Arkansas Med. Society....................... 231
" East Line Med. Society ..................... 20

Pneumonia, Treatment of............................ 325
Puerperal Eclampsia ................................ 192
Physicians' Mutual Benefit Association ...39, 68, 71, 134, 164, 270, 307, 338.
Pathology and Treatment of Chronic Cervical Endometritis 139
Placenta Prævia...................................... 158
President Wooten's Address......................... 157
Painless Extraction of Teeth........................ 168
Points in Medical Ethics............................ 56
Psoriasis, ℞ for.................................... 426
President Paine's address to members T. S. M. A..........334, 341
Priority in the use of Salol........................ 412
Public Trial of Surgical Skill...................... 419
Quarantine on the Rio Grande........................ 88
Quarantine Conference .............................. 336
Revision of U. S. Pharmacopœia...................... 420
"Rorick System of Anal Surgery" the................. 383
Regulating the Practice, etc........................ 346
Ringworm, ℞ for .................................... 163
Right Man in Right Place, the....................... 136
Reward, A........................................... 87
Railway Corporations, etc........................... 186
Railway "Boss" Surgeons and the Medical Profession...... 515
Successful Vaginal Hysterectomy..................... 248
Remarks on Uncomplicated Wounds and Ulcers.......... 405
Senator Plumb's bill................................ 178
Sensation in Medical Circles, A..................... 179
Society Notes........19, 68, 127, 164, 208, 264, 297, 334, 369, 440
Substitution by Druggists........................... 346
Swearingen, Dr. R. M., Biography of................. 486
Surgeon General M. H. S. and Journal A. M. A........ 347
Supreme Court Decision on Regulating the Practice, etc.. 346
Seborrhœa Sicca, ℞ for.............................. 426
Still-Born ......................................... 417
San Antonio Meeting of Texas State Medical Association 415
Texas State Med. Assoc., Officers....................88–9, 415
" " " " Transactions..............30, 32, 134
" " " " Notice to Members............ 414
" " " " San Antonio Meeting of....... 415
" " " " Proceedings of 21st An. Session 442
Treatment of Pneumonia.............................. 325
The Metric System................................... 503
Transplantation of Nerve from Rabbit to Man......... 33
The Big Meeting..................................... 428
The Old Oaken Bucket................................ 427
The Problem of Constipation Solved.................. 382
Travis County Medical Society.......................127, 212, 369

The Late Meeting—Texas State Medical Association .... 481
There's a Good Time a Coming........................ 377
Taxing Dentists.................................... 376
The Food in Acute Disease............................ 213
The President's Address to Members.................. 341
Traumatic Tetanus .................................... 12
Treatment of Whooping Cough........................ 17
Transactions Texas State Medical Association......30, 32, 86, 134
The Bulletin Racket.................................... 32
Treatment of Inflammation of Ileo Cœcal Valve......... 72
The Profession and the Public.......................... 75
The Texas Medical College............................ 133
The Texas Medical College, Equipment of............. 123
The Quack Must Go.................................... 129
The Hog as an Article of Food......................... 260
The Southern Surgical and Gynecological Society....... 264
Treatment of Yellow Fever............................ 171
The New Orleans Medical and Surgical Journal......... 177
Typhlitis.............................................. 281
The Hog: Bacon, Battles and By-gone Days............ 293
The Use of Pepsin in the Local Treatment of Diphtheria and Membranous Croup......................... 302
The Coming Meeting Texas State Medical Association.... 305
Texas Pharmacy Bill—Full Text........................ 518
Uncomplicated Ulcers and Wounds.................... 405
Vaginal Extirpation of Uterus......................... 74
Vesical and Urethral Calculi, fifteen cases............... 1
We Do Our Own Singing.............................. 313
We Take Our Hat Off.................................. 221
Weill's Disease........................................ 74
Yellow Fever, Treatment of........................... 171
Yellow Fever........................................... 77
Yerba Loco............................................ 103

